# An Institution-Based Assessment of Students' Hand Washing Behavior

**DOI:** 10.1155/2019/7178645

**Published:** 2019-12-27

**Authors:** Grace M. Mbouthieu Teumta, Loveline L. Niba, Nkwatoh Therese Ncheuveu, Mary-Teresia Ghumbemsitia, Paul O. B. Itor, Paul Chongwain, Lifoter K. Navti

**Affiliations:** ^1^Department of Microbiology, Catholic University of Cameroon (CATUC), P.O. Box 782, Bamenda, Cameroon; ^2^Department of Biochemistry, Catholic University of Cameroon (CATUC), P.O. Box 782, Bamenda, Cameroon; ^3^Faculty of Science Research Group, Catholic University of Cameroon (CATUC), P. O. Box 782, Bamenda, Cameroon

## Abstract

**Background:**

Hand hygiene is cost-effective and has been recognized as an effective measure in the control of communicable diseases. The main aim of this study is to evaluate the hand washing knowledge, practices, and skills of students in both private and public institutions of higher learning.

**Methods:**

This was an institution-based cross-sectional study that included a mix of 577 university students from different disciplines (49.6% males and 50.4% females) with a mean age of 21.8 ± 3.5 years. Departments were selected at random, and the quota sampling technique was used to select the study participants. Hand washing knowledge, practices, and skills scores were assessed using a structured questionnaire. The differences in scores were further quantified across different factors using multiple quantile regression analysis.

**Results:**

The prevalence of hand washing with soap was 10.7%. Also, the majority of the study participants (75.2%) had a poor hand washing practices score. Age ≥29 years was associated with a 6.3% lower median hand washing knowledge score (*p*=0.039). Also, being in the public institution was significantly associated with 6.3%, 36.0%, and 10.0% lower median hand washing knowledge (*p*=0.021), practices (*p* < 0.001), and skills (*p*=0.025) scores, respectively. In addition, being a medical student (*p*=0.029) and washing hands ≥ six times a day (*p*=0.008) were significantly associated with an increase in the median hand washing knowledge score of 6.3% and 18.7%, respectively.

**Conclusions:**

Educational interventions need to be carried out to create awareness of the importance of hand washing and also to enhance the hand washing knowledge, practices, and skills of university students.

## 1. Introduction

The transmission of communicable diseases continues to be a public health concern, and the physical contact through contaminated hands is a very important mode of transmission. Hand washing, which is a component of hand hygiene, is cost-effective and convenient and has been shown to be an effective practice in infection control activities [1]. There is undisputed evidence that when hands are properly washed under running water with soap lather, rubbed while washing, rinsed, and dried, the spread of a viral infection such as influenza [2] and bacterial diseases such as pneumonia and diarrhea [3] can be halted. For instance, a recent systematic review indicates that hand washing reduces the risk of diarrheal diseases by 40% [4]. A meta-analysis also indicates that washing hands reduces the risk of shigellosis by 59% and that failure to wash hands increases the risk of diarrheal diseases by 1.8 times [5]. Poor hand washing can result in the spread of other diseases such as cholera, hepatitis A, and acute gastroenteritis [6]. In recognition of the importance of hand washing, the WHO launched an annual global initiative, “SAVE LIVES: clean your hands,” in 2009 to create awareness of the importance of hand hygiene in healthcare [7].

Despite these efforts and the wealth of data showing the benefits of proper hand washing and its simplicity and efficacy, the global hand hygiene compliance has not been satisfactory. The global prevalence of washing hands with soap is 19%, and that of the African region is even lower at 14% [4]. Also, data on some African countries indicate a prevalence of 13%, 15%, and 8% for Ghana, Kenya, and Burkina Faso, respectively [4]. In addition, available data on Cameroon indicates that good hand washing practices were reported in less than half of dental professionals [8]. Some factors that could explain the inappropriate hand washing practices, especially among adolescents in low-income settings, are poor scientific knowledge, absence of clean running water [9], and lack of awareness and practice in hand washing [10]. Also, the efforts to improve hand hygiene can be compromised by cultural and religious beliefs in sub-Saharan African countries [11].

There are limited data on hand washing knowledge and practices among students in school settings in Cameroon. The spread of infectious diseases can occur faster in school settings because of high numbers of students, and the university community is not exempted. Recent reports show that school health can be protected during an epidemic if appropriate hand washing practices are carried out [12, 13]. There is evidence that students can overestimate their knowledge and compliance to hand hygiene practices [14]. This poor self-assessment can translate into an unsatisfactory compliance later in professional practice [15], especially among those involved in healthcare. Thus, the school setting becomes important and can provide a good avenue for community efforts to promote good hand hygiene practices including hand washing [16].

This study sets out to assess the hand washing knowledge, practices, and skills of students of different disciplines in two higher institutions of learning.

## 2. Materials and Methods

### 2.1. Study Participants

An institution-based cross-sectional study was carried out in two universities (one private and one public) in Bamenda, which is the capital of the North West Region of Cameroon. The sampling procedure was in two steps: Firstly, four departments were randomly selected in each institution. Secondly, a quota sampling technique was used to select students within each department who are either in the first, second, third, or fourth year of university studies following an arrangement with the respective departmental heads. All students were approached in their lecture halls as soon as lectures/tutorials were over. Since the students were selected from different departments in the two institutions, they were classified in the following groups (based on the faculty that hosts each degree programme) during the analysis: Social/Management sciences, Science/Engineering, and Medicine/Nursing. Consent information and the objectives of the study were given to the students, and this was followed by administration of the study questionnaire.

A total of 702 questionnaires were administered to the selected students. 577 (82.2%) completely filled questionnaires were returned. The analysis included 577 students (49.6% males and 50.4% females). There was no sex discrimination in the selection of study participants.

### 2.2. Ethical Considerations

Ethical/administrative clearance was obtained from the North West Regional Delegation of Public Health, Cameroon. Also, permission was obtained from establishment heads within each institution. All participants gave written informed consent before the study commenced.

## 3. Data Collection

### 3.1. Study Questionnaire

Data were collected (between January and April 2019) using a self-administered structured questionnaire by the principal investigator (G.M.M.T.), who met the students in their lecture rooms immediately after lectures. Before data collection, this instrument was pretested in a group of 24 students in a private institution of higher learning, which was not part of this study. This questionnaire was designed using previous reports [16, 17], and no validity tests were carried out. The questionnaire was divided into the following parts.

In the first part, the participants reported general information, which included age, gender, and department, and they responded to basic questions on hand washing.

The second part assessed hand washing knowledge using eight questions. The third part was on hand washing practices, and this aspect was assessed using 20 questions. The last part assessed hand washing skills of the study participants using 10 questions. The participant's score of each aspect of hand washing (knowledge, practices, and skills) was calculated by dividing the score obtained by each study participant in that aspect by the number of questions and multiplying by a hundred. Furthermore, the participants were classified based on their scores of the different aspects of hand washing as follows: poor, average, and good after having scores of ≤49.9%, 50.0–69.9%, and ≥70.0%, respectively.

In this study, hand washing was considered as the mechanical process of washing hands with soap under running clean water [16].

### 3.2. Statistical Analysis

R version 3.4.1., which includes the “quantreg” package [18], was used to perform all statistical procedures. The Kolmogorov-Smirnov (K-S) test was used to assess the distribution of continuous variables. Also, a descriptive analysis of the study population was performed, and values were reported as frequencies and percentages. The comparison of proportions was carried out using the chi-square test, and the *p* values were adjusted using the Bonferroni method. In addition, a comparison of median score of the different aspects of hand washing across groups was carried out using the Kruskal–Wallis test. After the unadjusted analysis, the differences in median score were further adjusted using multiple quantile regression analysis. In this regression analysis, the median score was modeled as dependent variable instead of the mean because the scores of the different aspects of hand washing (knowledge, practices, and skills) were not normally distributed. Furthermore, the results are easily understood because the regression coefficients reveal the difference in median score associated with each factor compared with a reference group. The model in the regression analysis was adjusted for design variables (institution and department) and also included all variables in the unadjusted analysis. The Powell Kernel approach was used to calculate the regression estimates of the median score and standard errors. A two-sided *p* value <0.05 was considered significant.

## 4. Results

A sample of 577 university students with a mean age of 21.8 ± 3.5 years was included in this study.  Table 1 shows that more than half (56.7%) of the students wash their hands ≥6 times a day, with girls having a significantly higher proportion than boys (*p*=0.018). Also, the prevalence of hand washing with soap and clean running water in the study population was 10.7%. In addition, 47.8% of the study participants indicated that the reason for skipping hand washing is forgetfulness. 1.9% and 75.2% of the study participants had good and poor hand washing practices scores, respectively.

The median (min–max) hand washing knowledge, practices, and skills scores of the full sample were 62.5 (0.0–100), 21.3 (0.0–75.0), and 49.7 (0.0–100), respectively. The unadjusted analysis in Table 2 shows that older students (≥29 years) had significantly (*p* < 0.001) lower median hand washing knowledge and practices scores when compared with the younger students (17–22 years). Also, the medical students (Medicine/Nursing) had a more than 10% increase in median hand washing knowledge score when compared with the rest of the study participants. However, they had a significantly lower (*p* < 0.001) median hand washing practice score. There were no significant gender differences in median hand washing knowledge, practices, and skills scores (*p* > 0.05). In addition, there was a 36.9% difference in median hand washing practice score between students of the private university and those of the public university. Students who washed their hands ≥6 times a day had significantly higher median hand washing knowledge, practices, and skills scores (*p* < 0.05) compared with those who wash their hands ≤2 times a day.

Table 3 shows the quantile regression estimates for the association between selected factors and scores of the different aspects of hand washing. When compared with their respective reference groups, age ≥29 years and being a student of a public university were significantly (*p* < 0.05) and negatively associated with median hand washing knowledge score. The older students (≥29 years) had a 6.3% lower median hand washing knowledge score when compared with the younger students (17–22 years). Also, medical students (Medicine/Nursing) had a 6.3% higher median hand washing knowledge score when compared with the rest of the students (*p*=0.029). In addition, students who wash their hands ≥6 times a day had a 18.7% higher median hand washing knowledge score when compared with those who wash their hands ≤2 times a day (*p*=0.008). These students also had a higher median hand washing skills score. However, it was not statistically significant (*p* > 0.05). Furthermore, when compared with the students of the private university, their peers in the public university had 6.3%, 36.0%, and 10.0% lower median hand washing knowledge, practices, and skills scores, respectively (*p* < 0.05). Thus, the factors with the largest significant relationship with the scores were age ≥29 years, being a medical student, being a student in a public university, and washing of hands ≥6 times a day for hand washing knowledge score and being in a public university for hand washing practices and skills scores.

## 5. Discussion

This study describes the current state of hand washing behavior in a sample of university students in Cameroon for the first time. The study evaluated the hand washing knowledge, practices, and skills of students from both private and public universities. The analysis confirms that age was inversely associated with hand washing knowledge score, and being in a public university was also inversely associated with hand washing knowledge, practices, and skills scores. In addition, the study confirms that being a medical student (Medicine/Nursing) was positively associated with hand washing knowledge score, and washing of hands ≥6 times a day was positively associated with hand washing knowledge, practices, and skills scores.

Older age was significantly associated with a lower median knowledge score. Similar findings have been observed in studies carried out in Ghana [17] and Bangladesh [19], which used different approaches of assessing hand washing knowledge and practices. These studies indicated that older students had lower hand hygiene knowledge and practices scores when compared with their younger peers. A previous report had indicated that the poor hand hygiene behavior among adults could be as a result of their busy lifestyle and a false belief that infectious diseases like diarrhea can only affect younger children [20]. However, in a previous study in Turkey, age was positively associated with knowledge score [16]. Also, a study among adolescents in Yemen indicated that younger children had higher odds of poor hand washing practice [21].

Our study reveals that there were no gender differences in hand washing knowledge, practices, and skills scores. This is in contrast to findings of other studies which recorded significant differences in mean hand washing knowledge and practices scores [17, 19] between males and females. When the frequency of hand washing was considered, this study revealed that there was a significant difference by gender. A significantly higher proportion of females washed their hands ≥6 times a day when compared with the males. This is in line with other studies which revealed that females are more likely to wash their hands frequently than males [22, 23]. A study reported that a high compliance to hand hygiene among females could be as a result of their tendency to practice socially acceptable behavior [24]. Also, males tend to ignore hand hygiene practices, especially when they are alone in the washroom or when they are in a hurry [25]. In addition, males can be discouraged from practicing hand hygiene by factors such as tiredness and hunger [26]. However, a recent report recorded better hand hygiene practices among men [27].

Also, this study indicated that being a medical student (Medicine/Nursing) was associated with a higher median hand washing knowledge score. A recent study among nursing students indicated that the majority of the study participants had good knowledge of hand hygiene and this translated into a positive attitude towards hand hygiene compliance [28]. This could be as a result of the high hygienic standards expected from these students who are usually under supervision in hospital settings. A report indicated that medical students are knowledgeable and more likely to practice hand hygiene effectively because they are aware of the risks of noncompliance to hand washing practice [29]. However, a study among undergraduate medical students in Kampala indicated that more than 50% of the study participants had poor knowledge on hand hygiene. More than 40% of the study participants were not aware of the importance of hand washing, and approximately 90% of them indicated that there was lack of clean running water in hospital wards during their clinical activities [30]. These could have acted as barriers to hand hygiene practices and compliance.

Our study further reveals that being in a public university was associated with lower median hand washing knowledge, practices, and skills scores. During the study period, it was observed that wash rooms for students in the public university were far from lecture halls, and the availability of clean running water on campus was not regular. On the contrary, the private institution was a smaller learning environment with a well installed water supply system including bore holes. In addition, there are posters which indicate how to use the facilities in the wash room. These posters and easy access to wash room facilities could encourage good hygienic practices and hand washing, in particular among students of the private institution. For instance, a report in the US indicated that the use of soap in hand washing was more in wash rooms that had hand washing instructions on posters than wash rooms with no posters [31]. However, we did not investigate the contribution of these items to hand washing behavior of the students included in this study.

Washing hands ≥6 times a day was associated with a higher median hand washing knowledge score. When students are knowledgeable of the benefits of hand washing, they are likely to wash their hands frequently. In a recent study, knowledge was significantly associated with hand hygiene practice in a sample of nursing students [28]. However, the sample size of the study was small. In our study, 56.6% of the participants indicated that they wash their hands ≥6 times a day. This is lower when compared with a study in Turkey in which the majority (72.6%) of the study participants wash their hands ≥6 times a day [16]. The participants in this study indicated the lack of clean running water on campus, dirty wash room facilities, forgetting to wash hands, and wash rooms being far away as the reasons for skipping hand washing. These and other reasons identified in other studies like lack of soap, laziness, lack of awareness on hand hygiene importance [30], and cleanliness of wash room [22] could explain the disparity in the proportions of students with a high frequency of hand washing (or hand washing behavior) between our sample and that of the Turkish study. These could also explain why the majority (75.2%) of our study participants had a poor hand washing practice score. The lack of soap could further explain the low prevalence of hand washing with soap observed in this study and also explains why the majority of the students indicated that they wash their hands only with clean water.

Our study had limitations worth mentioning. The analysis included self-reported data on hand washing behavior of students. There could have been bias in reporting as some students are likely to over report their hand washing behavior [14]. Also, we did not independently observe if the students washed just one hand or both hands. Our study included a small sample, and the findings may not adequately reflect the hand washing behavior of the entire university student population of Cameroon. However, the data included a mix of students in different disciplines from both private and public institutions. Despite these limitations, we have quantified the differences in median scores of the different aspects of hand washing across several factors using quantile regression analysis for a better appreciation.

## 6. Conclusions

The findings of this study reveal that the majority of the students had poor hand washing practice score and the prevalence of hand washing with soap is low. The study also highlights that the physical environment of hand washing needs to be conducive, especially in the public institution. In line with this, the provision of soap, regular availability of clean running water, and regular hygienic sanitation of wash room facilities are a necessity. These could encourage students to wash hands frequently. Random visits to the wash rooms and observations of hand washing could help in understanding the hand hygiene behavior of students in future studies. Also, educational interventions need to be implemented to enhance the hand washing knowledge, practices, and skills of the students.

## Figures and Tables

**Table 1:** Descriptive characteristics of the study population (*N* = 577).

Variables	Gender	
	Male (*N* = 286)	Female (*N* = 291)
Age (years)		
17–22	172 (60.1)	210 (72.2)
23–28	97 (33.9)	72 (24.7)
≥29	17 (5.9)	9 (3.1)
Discipline		
Social/management sciences	137 (47.9)	152 (52.2)
Science/engineering	91 (31.8)	59 (20.3)
Medicine/nursing	58 (20.3)	80 (27.5)
Type of university		
Private	164 (57.3)	140 (48.1)
Public	122 (42.7)	151 (51.9)
Frequency of hand wash per day		
≤2 times	11 (3.8)	12 (4.1)
3–5 times	129 (45.1)	98 (33.7)
≥6 times	146 (51.1)^a^	181 (62.3)^a^
Hand hygiene habits		
Hand washing with soap and clean water	24 (8.4)	38 (13.1)
Hand washing with clean water only	253 (88.5)	237 (81.4)
Use of alcohol hand sanitizers	9 (3.1)	16 (5.5)
Reasons for skipping hand washing		
Wash room is far	51 (17.8)	51 (17.5)
Lack of clean running water	57 (19.9)	41 (14.1)
Dirty wash room facilities	55 (19.2)	46 (15.8)
Forgetting	123 (43.0)	153 (52.6)
Hand washing knowledge score		
Poor	57 (19.9)	59 (20.3)
Average	114 (39.9)	106 (36.4)
Good	115 (40.2)	126 (43.3)
Hand washing practices score		
Poor	213 (74.5)	221 (75.9)
Average	68 (23.8)	64 (22.0)
Good	5 (1.7)	6 (2.1)
Hand washing skills score		
Poor	107 (37.4)	99 (34.0)
Average	82 (28.7)	75 (25.8)
Good	97 (33.9)	117 (40.2)

^a^ *p*=0.018.

**Table 2:** Comparison of median scores across the different groups (*N* = 577).

Factors	Knowledge score		Practices score		Skills score	
	Median	*p* value^a^	Median	*p* value^a^	Median	*p* value^a^
Age (years)		<0.001		<0.001		0.624
17–22	65.9		34.0		51.0	
23–28	57.7		10.9		46.0	
≥29	51.3		9.3		46.7	
Gender		0.556		0.279		0.585
Male	60.9		27.3		48.0	
Female	62.5		14.5		51.8	
Discipline		<0.001		<0.001		0.337
Social/management sciences	59.5		12.0		46.4	
Science/engineering	62.5		44.1		50.8	
Medicine/nursing	73.0		11.9		55.4	
Type of university		0.002		<0.001		0.895
Private	66.4		45.8		48.9	
Public	58.3		8.9		51.1	
Frequency of hand wash per day		<0.001		0.043		0.012
≤2 times	42.2		12.5		38.6	
3–5 times	60.1		21.4		41.5	
≥6 times	62.5		23.1		53.6	

^a^Kruskal–Wallis test was used to compare the median scores across the different factors.

**Table 3:** Multiple quantile regression estimates indicating differences in hand washing knowledge, practices and skills scores across different factors (*N* = 577).

Factors	Knowledge score			Practices score			Skills score		
	Estimate	SE	*p* value	Estimate	SE	*p* value	Estimate	SE	*p* value
Intercept	50.0	7.6	<0.001	44.0	2.6	<0.001	30.0	12.8	0.020
Age (years)									
17–22	0			0			0		
23–28	−6.3	3.4	0.065	0.5	1.0	0.496	−10.0	4.4	0.025
≥29	−6.3	6.6	0.039	0.5	1.8	0.274	−10.0	11.1	0.368
Gender									
Male	0			0			0		
Female	0.0	2.6	1.000	0.5	0.8	0.544	0.0	3.9	1.000
Discipline									
Social/management sciences	0			0			0		
Science/engineering	0.0	3.1	1.000	1.5	1.0	0.091	10.0	4.8	0.036
Medicine/nursing	6.3	3.2	0.029	2.0	1.2	0.328	0.0	5.1	1.000
Type of university									
Private	0			0			0		
Public	−6.3	3.2	0.021	−36.0	1.1	<0.001	−10.0	4.5	0.025
Frequency of hand wash per day									
≤2 times	0			0			0		
3–5 times	12.5	7.3	0.086	1.0	2.3	0.683	10.0	12.4	0.420
≥6 times	18.7	7.1	0.008	1.5	2.3	0.537	20.0	12.3	0.104

The estimate associated with each category is the difference in median score compared with the reference category. SE, standard error.

## Data Availability

The data used to support the findings of this study are available from the corresponding author upon request.
